# Supplementary material for: Senescent-like macrophages mediate angiogenesis for endplate sclerosis via IL-10 secretion in male mice
Source: Nat Commun. 2024 Apr 5;15:2939. doi: 10.1038/s41467-024-47317-1 (PMC10997778; doi:10.1038/s41467-024-47317-1)
Supplement: Supplementary file 1 — Supplementary Information [file 41467_2024_47317_MOESM1_ESM.pdf]

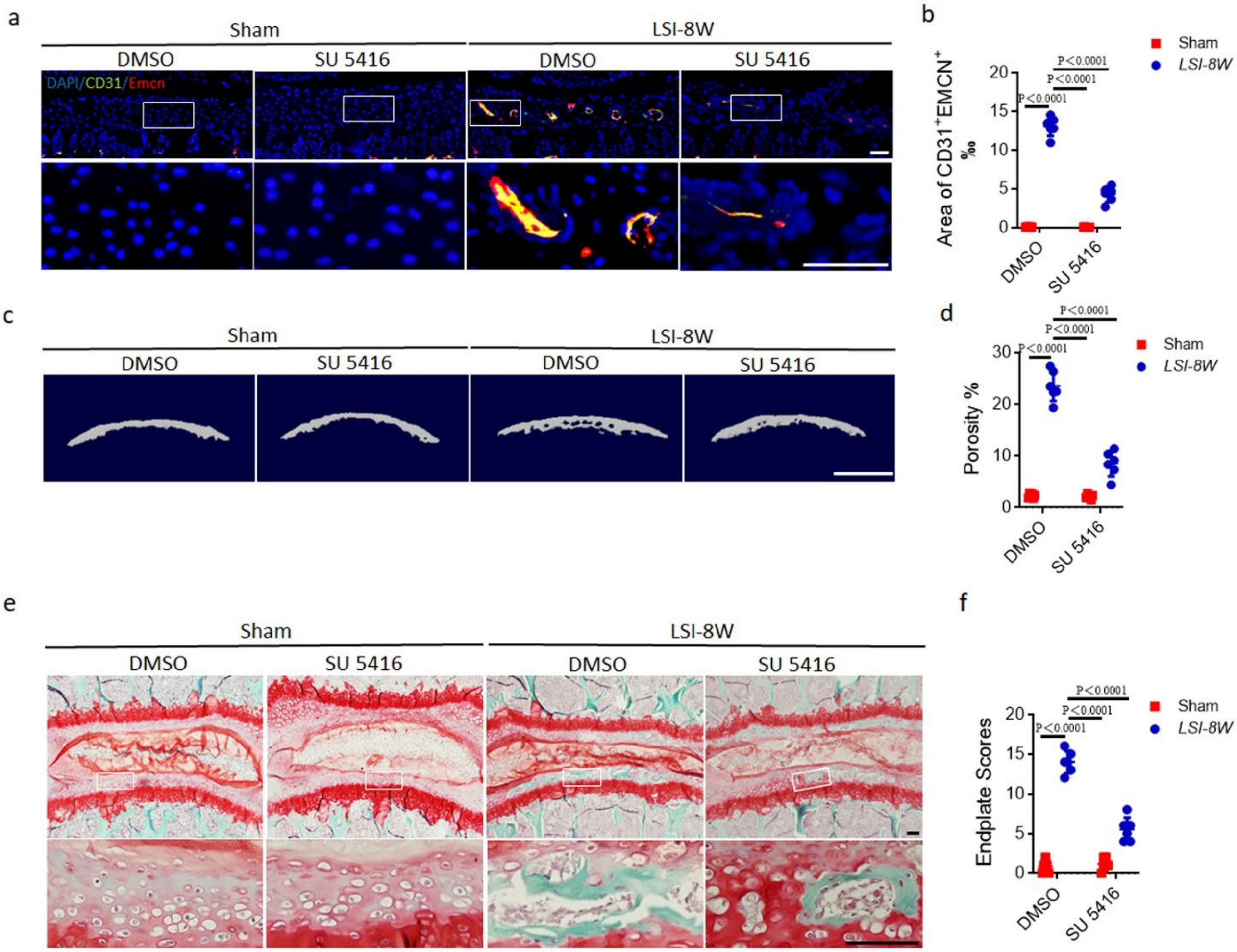

Supplementary Figure 1 VEGFR inhibitor blocks the angiogenesis and sclerosis of endplates

(a) Representative immunofluorescent images of $CD31^+$ (green), $Emcn^+$ (red) cells, and DAPI (blue) staining of the endplates at 8 weeks after LSI surgery or sham surgery. Scale bars, 50 µm. (b) Permillage of $CD31^+Emcn^+$ area of (a). (c) Representative three-dimensional µCT images of the endplates (coronal view) at 8 weeks after LSI surgery or sham surgery. Scale bars, 500 µm. (d) Quantitative analysis of the total porosity of (c). (e) Representative images of safranin O and fast green staining at 8 weeks after LSI or sham surgery, proteoglycan (red), and cavities (green). Scale bars, 50 µm. (f) Endplate scores of (e). n = 6 per group. Data are represented as means ± standard deviations, as determined by One-way ANOVA. Source data are provided as a Source Data file.

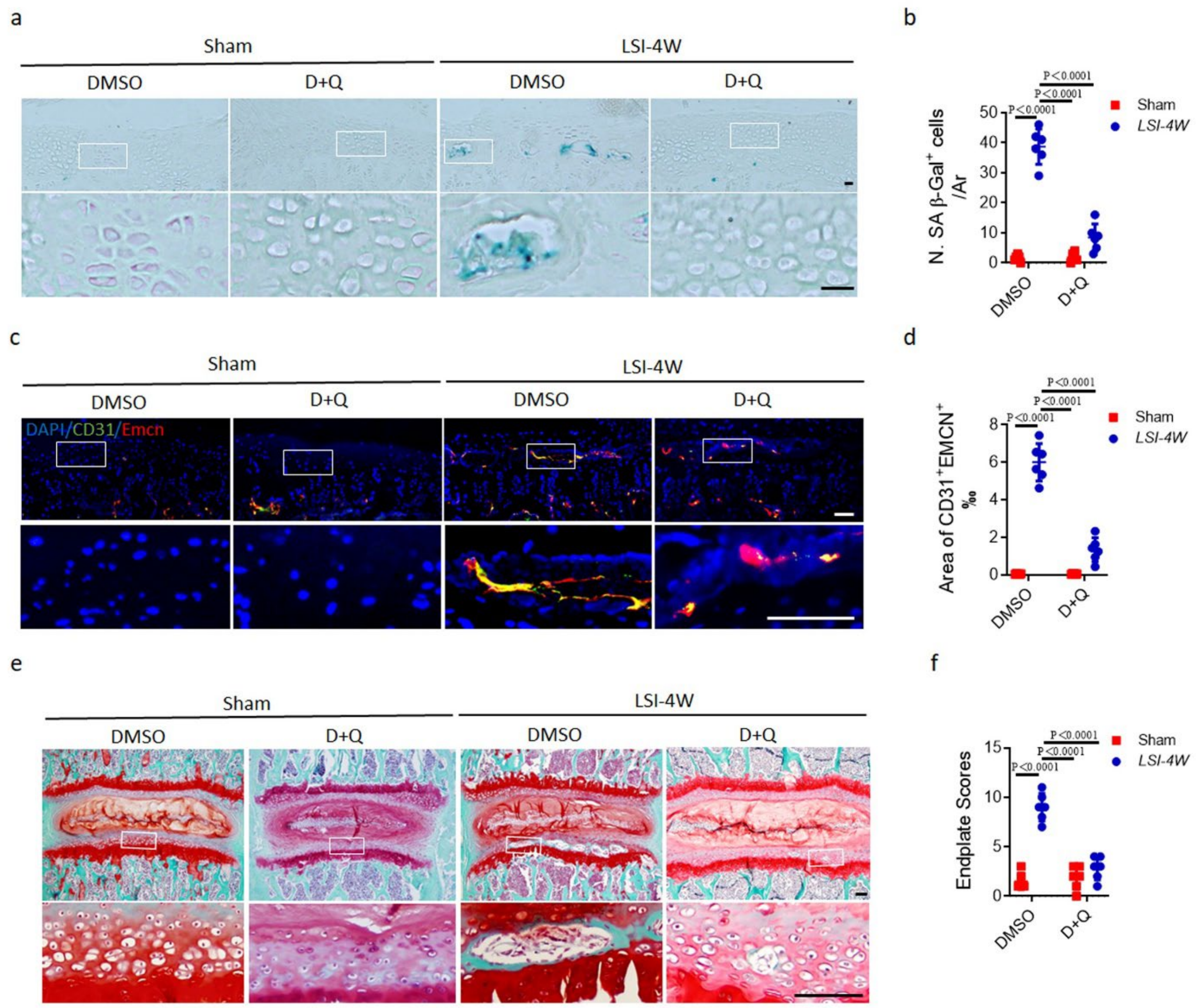

Supplementary Figure 2 The sclerosis of endplates is delayed by D+Q treatment in mice at 4 weeks after LSI surgery

(a) Representative images of coronal mouse caudal endplate sections of L4/5 stained for SA-βGal (green). Scale bars, 50 µm. (b) Quantitative analysis of (a). (c) Representative immunofluorescent images of CD31 (green), Emcn (red) staining, and DAPI (blue) staining of nuclei. Scale bars, 50 µm. (d) Permillage of $CD31^{+}Emcn^{+}$ area in the endplates of (c). (e) Representative images of safranin O and fast green staining, proteoglycan (red), and cavities (green). Scale bars, 50 µm. (f) Endplate scores as an indication of endplate degeneration based on safranin O and fast green staining. n = 6 per group. Data are represented as means ± standard deviations, as determined by One-way ANOVA. Source data are provided as a Source Data file.

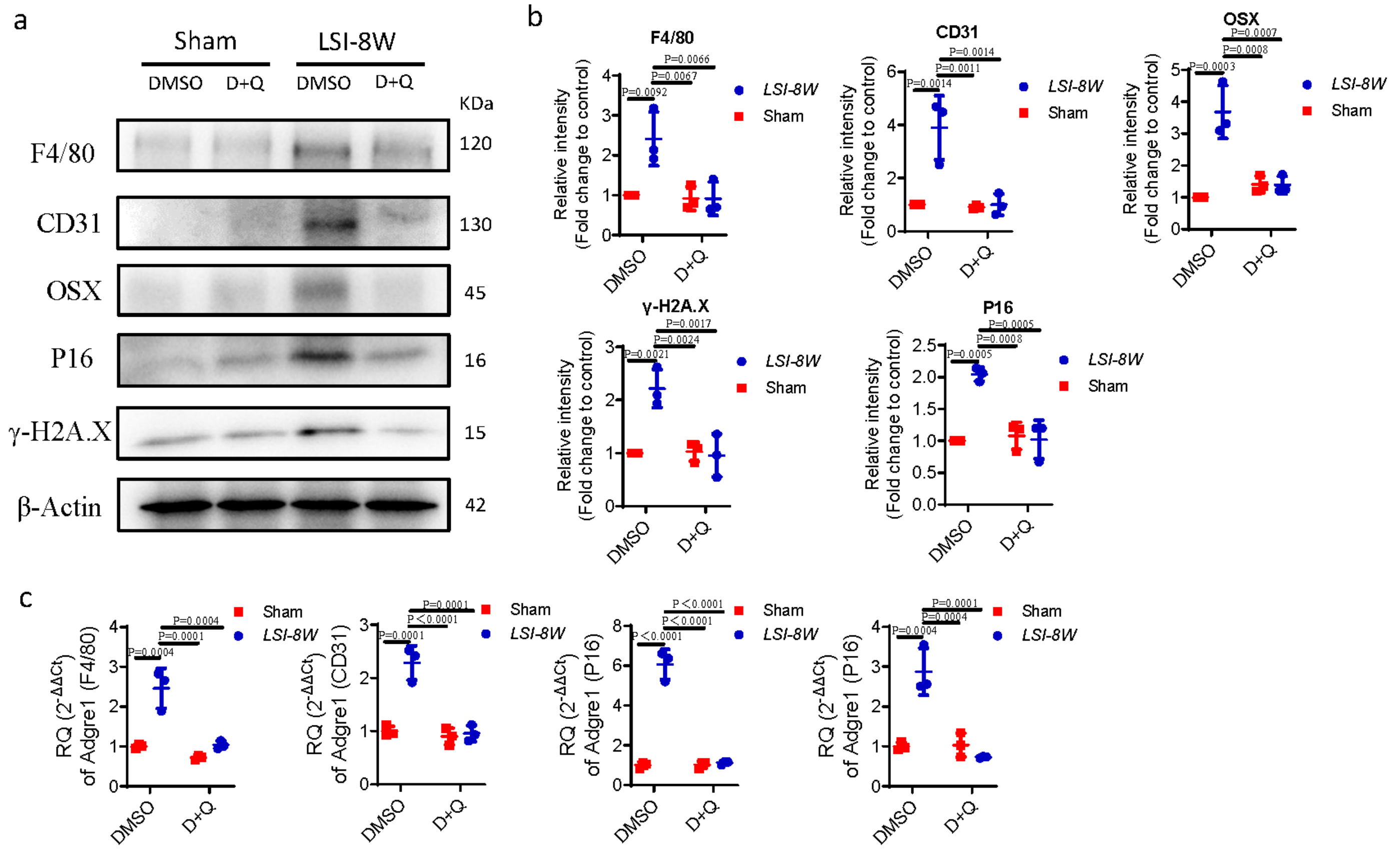

Supplementary Figure 3 (a) Western blots of F4/80, CD31, OSX, P16, and γ-H2A.X in the endplates at 8 weeks after LSI surgery with DMSO or D+Q treatment. (b) Quantitative analysis of (a). (c) QRT-PCR analysis of F4/80, CD31, OSX, and P16 in the endplates at 8 weeks after LSI surgery with DMSO or D+Q treatment. n = 3 per group. Data are represented as means ± standard deviations, as determined by One-way ANOVA. Source data are provided as a Source Data file.

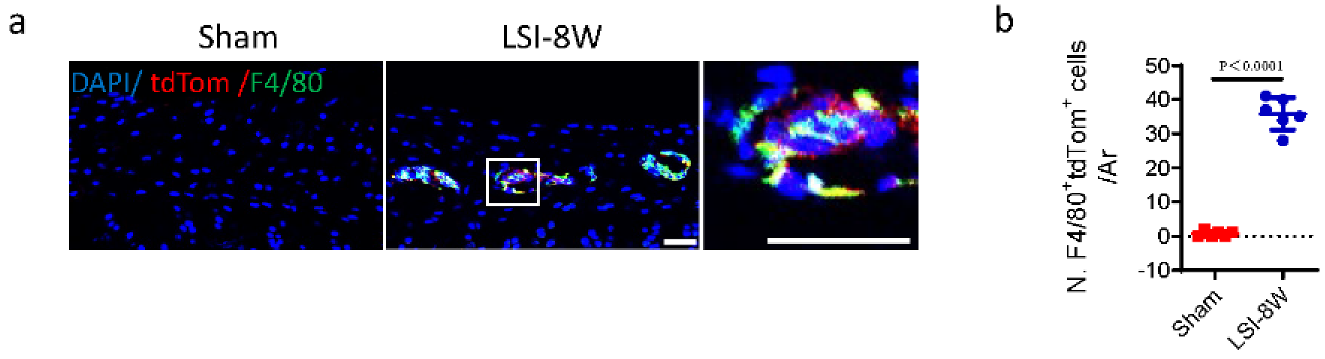

Supplementary Figure 4 The number of tdTom$^+$F4/80$^+$ cells increases in the endplates at 8 weeks after LSI surgery. (a) Representative immunofluorescent images of tdTom$^+$ (red), F4/80$^+$ (green) staining, and DAPI (blue) staining of nuclei. Scale bars, 50 µm. (b) Quantitative analysis of the number of F4/80$^+$tdTom$^+$ cells in the endplates of (a). n = 6 per group. Data are represented as means ± standard deviations, as determined by two-tailed Student's t-test. Source data are provided as a Source Data file.

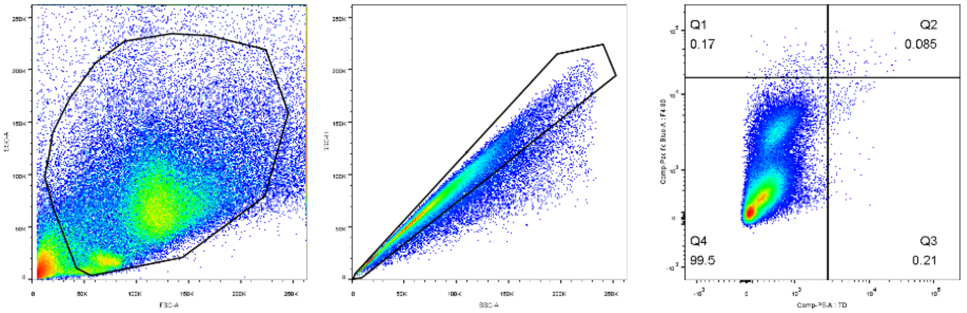

Supplementary Figure 5 The gating strategy for the flow cytometry analysis.

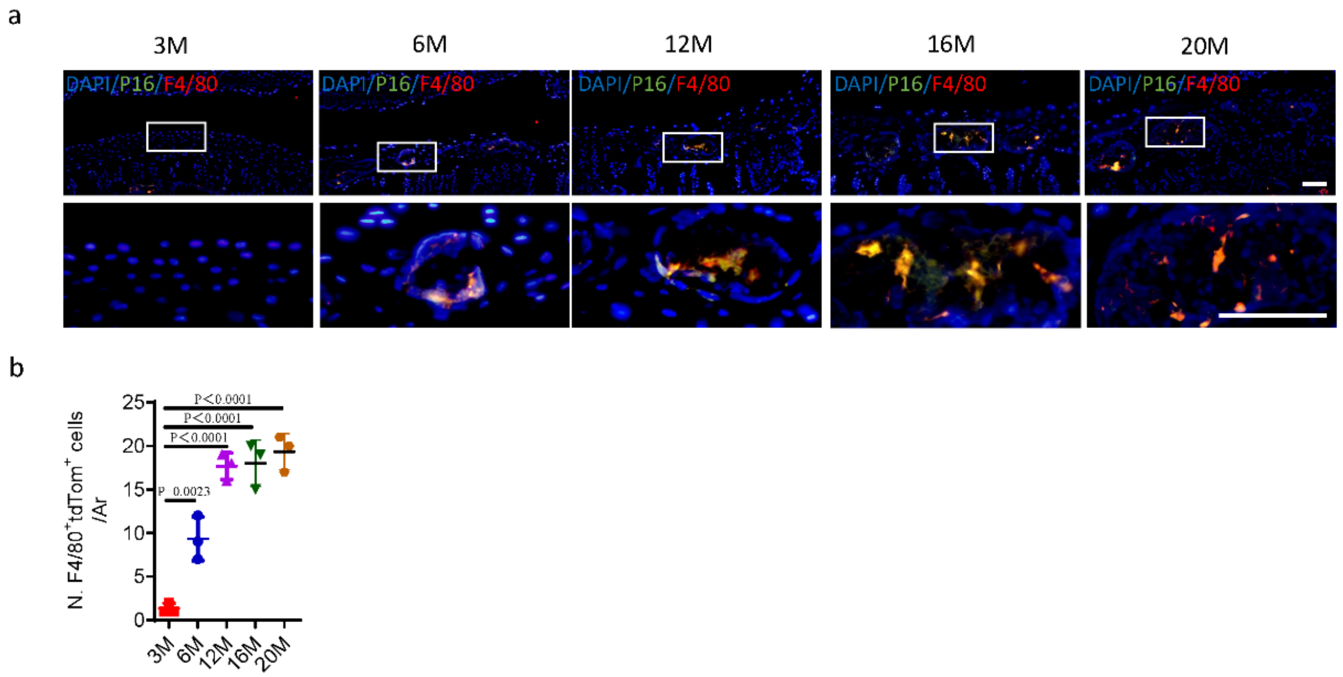

Supplementary Figure 6 Macrophages are already senescent upon infiltration into the endplates. (a) Representative co-immunofluorescent images of F4/80$^+$ (red) with P16$^+$ (green) staining in the endplates of 3, 6, 12, 16, and 20-month-old mice. (b) Quantitative analysis of (a). Scale bars, 50 µm. n = 3 per group. Data are represented as means ± standard deviations, as determined by One-way ANOVA. Source data are provided as a Source Data file.

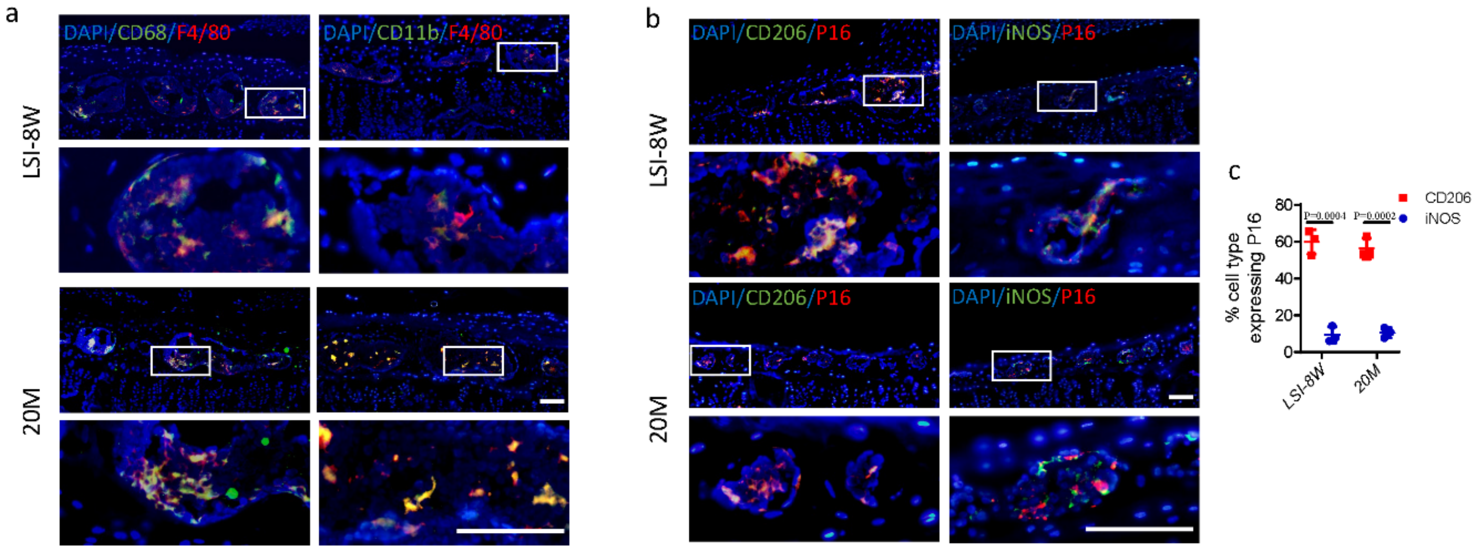

Supplementary Figure 7 Senescent-like macrophages express more M2 marker in the endplates of LSI surgery mice and aged mice. (a) Representative co-immunofluorescent images of F4/80 $^{+}$ (red) with CD68^{+} or CD11b^{+} (green) staining in the endplates at 8 weeks after LSI surgery or in 20-month-old mice. (b) Representative co-immunofluorescent images of P16^{+} (red) with CD206^{+} or iNOS^{+} (green) staining in the endplates at 8 weeks after LSI surgery or in 20-month-old mice. (c) Quantitative analysis of (b). Scale bars, 50 µm. n = 3 per group. Data are represented as means ± standard deviations, as determined by One-way ANOVA. Source data are provided as a Source Data file.

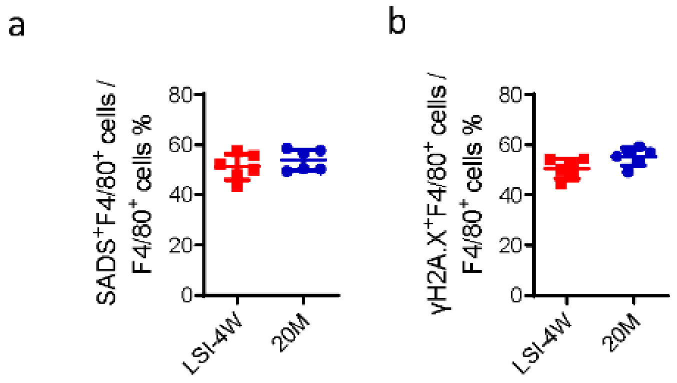

Supplementary Figure 8 (a,b) The percentage of the $SADS^{+}/F4/80^{+}$ (a) or $\gamma H2A.X^{+}/F4/80^{+}$ (b) double-positive cells over $F4/80^{+}$ cells in the endplates at 4 weeks after LSI surgery or in 20-month-old mice. n = 6 per group. Data are represented as means ± standard deviations. Source data are provided as a Source Data file.

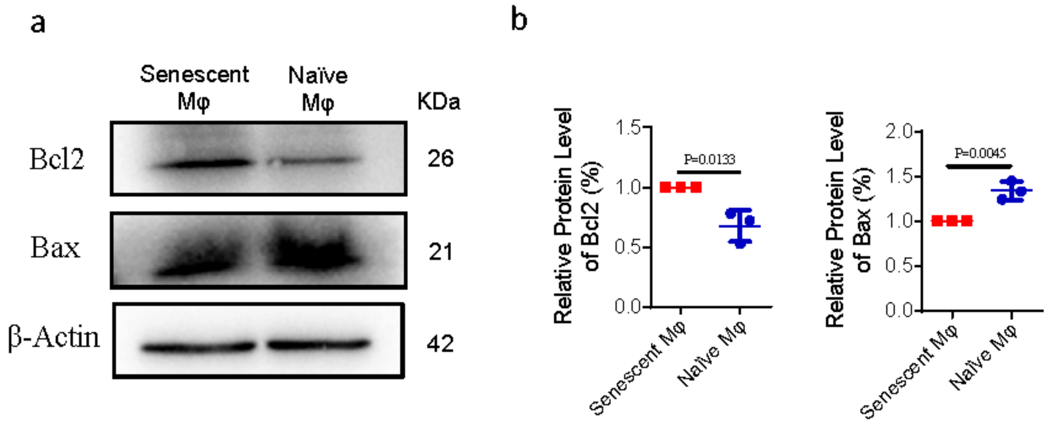

Supplementary Figure 9 The increased anti-apoptotic activity in senescent bone marrow-derived macrophages. (a) Western blots of the Bcl2 and Bax in bone marrow-derived macrophages administrated with 100 μM H_2O_2 or PBS for 4 hours. (b) Quantitative analysis of (a). n = 3 per group. Data are represented as means ± standard deviations, as determined by two-tailed Student's t-test. Source data are provided as a Source Data file.

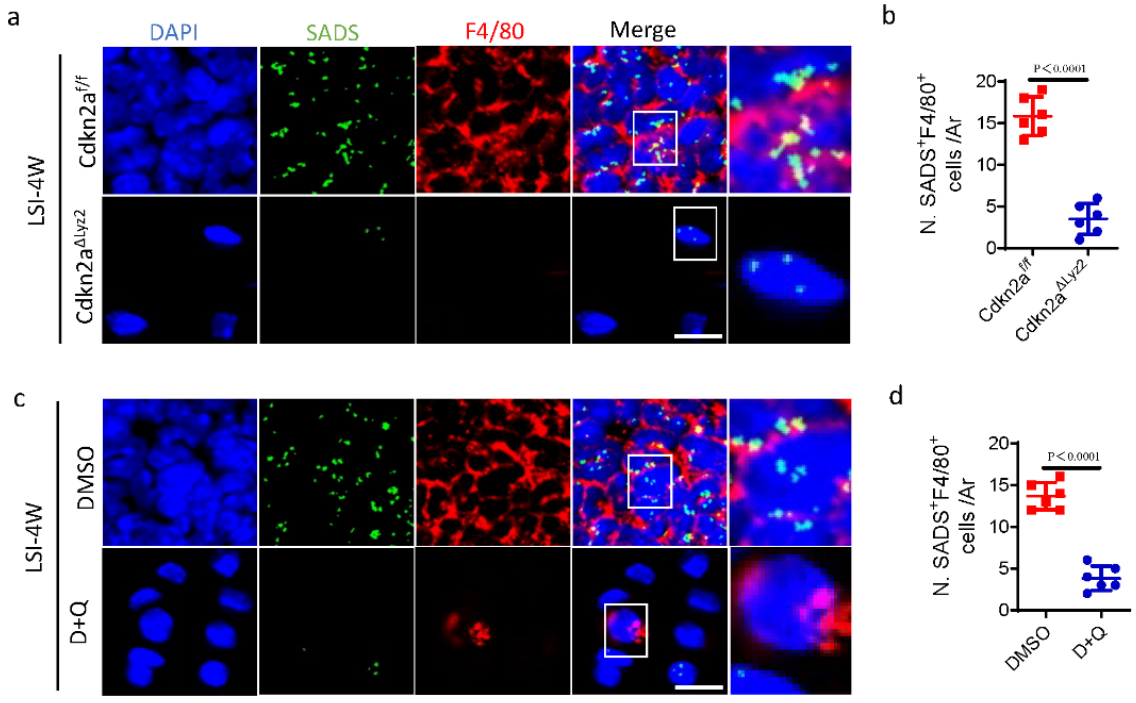

Supplementary Figure 10 The LSI-induced increase in the number of F4/80$^+$SADS$^+$ cells (≥ 4 SADS/cell) in the endplates is diminished by D+Q treatment or conditional knockout of Cdkn2a in Lyz2$^+$ cells. (a, c) Representative images of senescence-associated distension of satellites (SADS, green), F4/80 (red), and DAPI (blue) staining of nuclei in the endplates at 4 weeks after LSI surgery by performing simultaneous immunofluorescent staining and immune-fluorescent in situ hybridization (FISH). Scale bars, 10 μm. (b, d) Quantitative analysis of the number of F4/80$^+$SADS$^+$ cells (⩾4 SADS/cell) in the endplates of (a, c). n = 6 per group. Data are represented as means ± standard deviations, as determined by two-tailed Student's t-test. Source data are provided as a Source Data file.

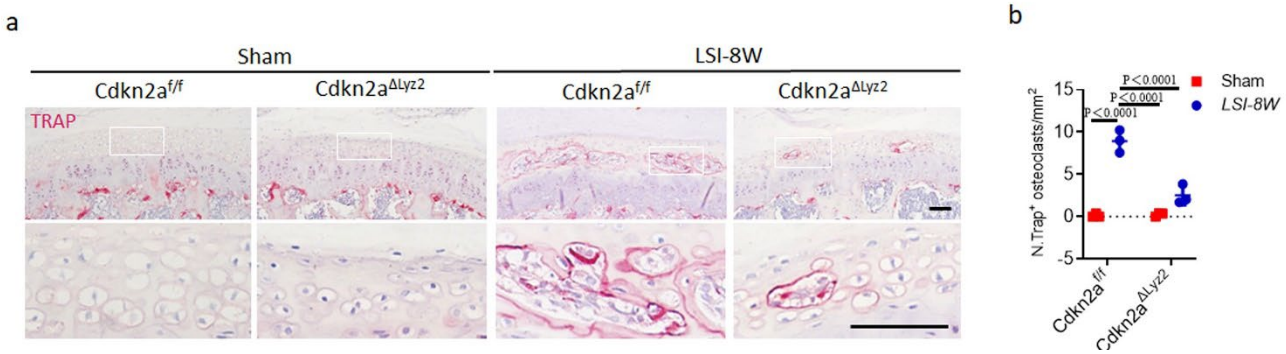

Supplementary Figure 11 The conditional knockout of Cdkn2a decreases the number of osteoclasts in the endplates of LSI mice. (a) Representative images of tartrate-resistant acid phosphatase (TRAP) (magenta) at 8 weeks after LSI or sham surgery in Cdkn2a$^{\Delta Lyz2}$ mice or Cdkn2a$^{f/f}$ mice. Scale bars, 50 μm. (b) Quantitative analysis of the number of TRAP^{+} cells in the endplates. n = 3 per group. Data are represented as means ± standard deviations, as determined by One-way ANOVA. Source data are provided as a Source Data file.

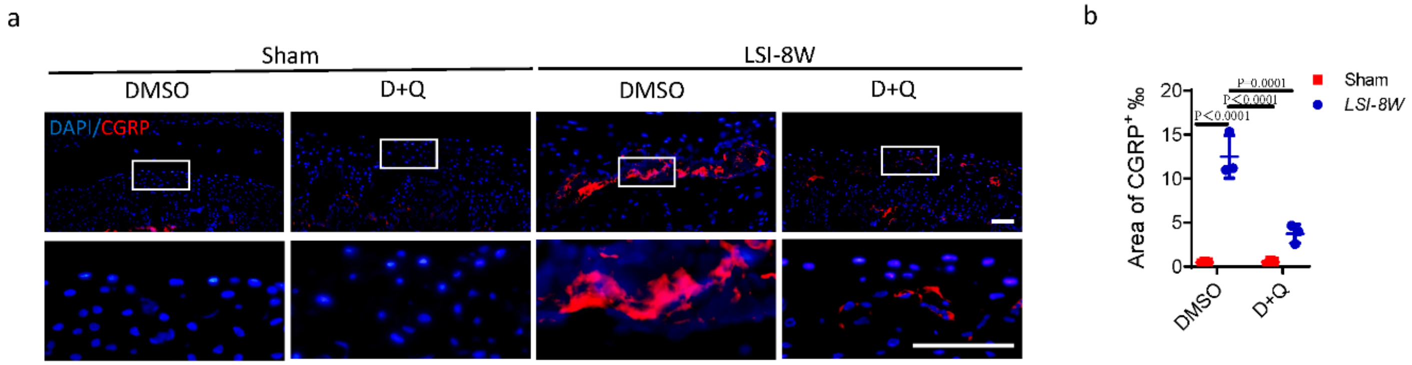

Supplementary Figure 12 D+Q treatment decreases the density of CGRP$^+$ sensory nerves in the endplates of LSI surgery mice. (a) Representative images of immunofluorescent staining of CGRP$^+$ sensory nerves (red) and DAPI (blue) staining of nuclei in the endplates. Scale bars, 50 μm. (b) Permillage of CGRP$^+$ area in the endplates of (a). n = 3 per group. Data are represented as means ± standard deviations, as determined by One-way ANOVA. Source data are provided as a Source Data file.

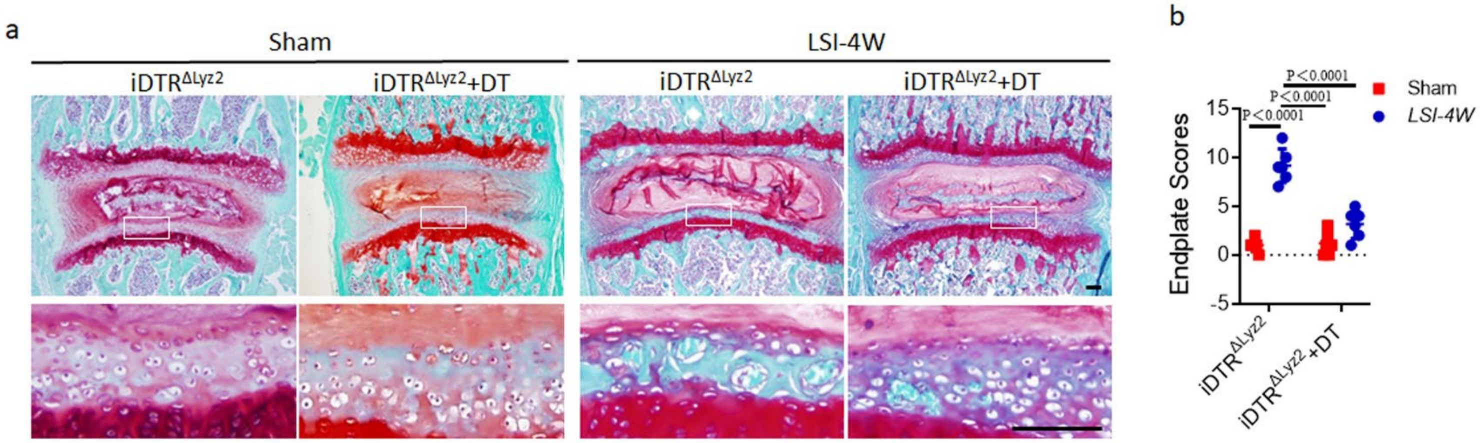

Supplementary Figure 13 DT injection inhibits LSI-induced endplate morphological degeneration at 4 weeks in iDTR$^{\Delta Lyz2}$ mice (a) Representative images of safranin O and fast green staining of the endplates. Scale bars, 50 µm. (b) Endplate scores of (a). n = 6 per group. Data are represented as means ± standard deviations, as determined by One-way ANOVA. Source data are provided as a Source Data file.

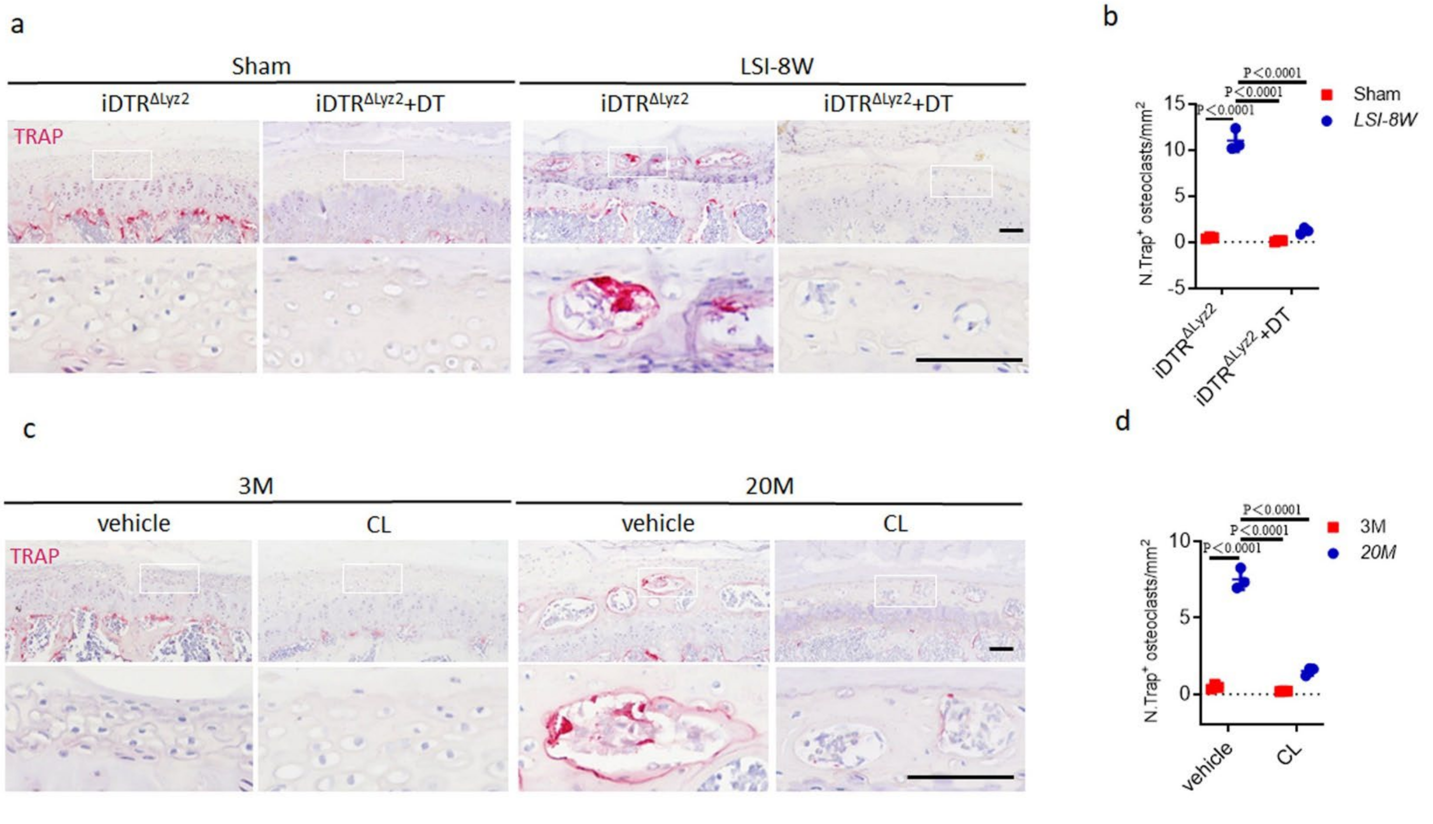

Supplementary Figure 14 The macrophage deletion decreases the number of osteoclasts in the endplates of LSI mice. (a) Representative images of tartrate-resistant acid phosphatase (TRAP) (magenta) at 8 weeks after LSI or sham surgery in iDTR$^{\Delta Lyz2}$ mice treated with DT or vehicle. Scale bars, 50 μm. (b) Quantitative analysis of the number of TRAP^{+} cells in the endplates of (a). (c) Representative images of TRAP (magenta) in 3-month-old mice or 20-month-old mice treated with CL or vehicle. Scale bars, 50 μm. (d) Quantitative analysis of the number of TRAP^{+} cells in the endplates of (c). n = 3 per group. Data are represented as means ± standard deviations, as determined by One-way ANOVA. Source data are provided as a Source Data file.

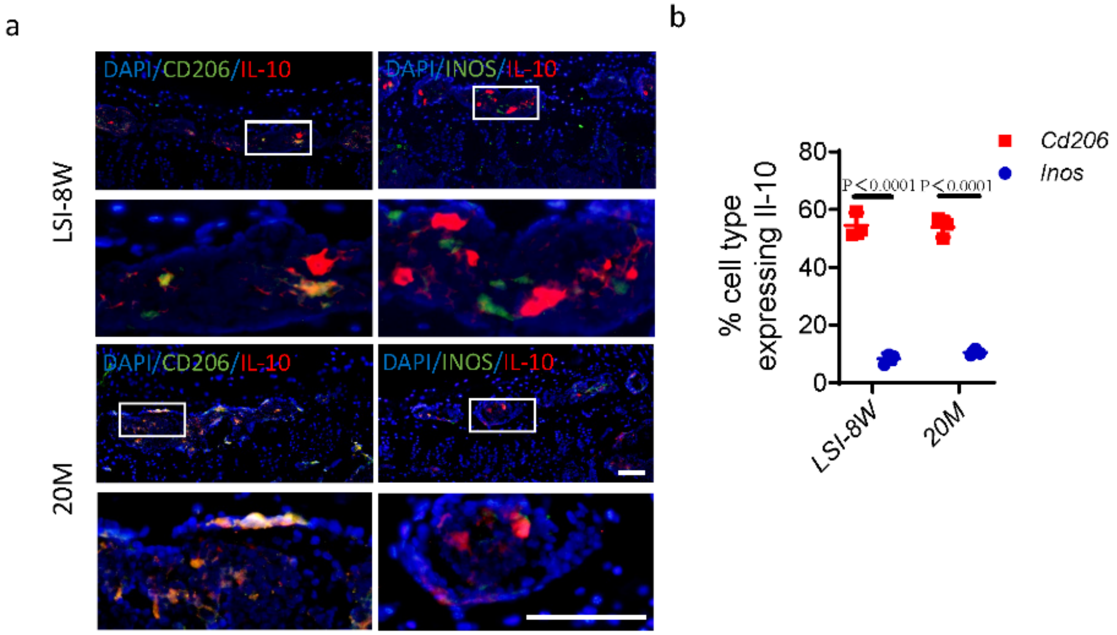

Supplementary Figure 15 The cells expressing IL-10 mainly co-localized with CD206$^+$ cells rather than iNOS$^+$ cells in the endplates of LSI surgery mice or aged mice. (a) Representative co-immunofluorescent images of IL-10$^+$ (red) with CD206$^+$ or iNOS$^+$ (green) staining in the endplates at 8 weeks after LSI surgery or in 20-month-old mice. (b) Quantitative analysis of (b). Scale bars, 50 µm. n = 3 per group. Data are represented as means ± standard deviation, as determined by One-way ANOVA. Source data are provided as a Source Data file.

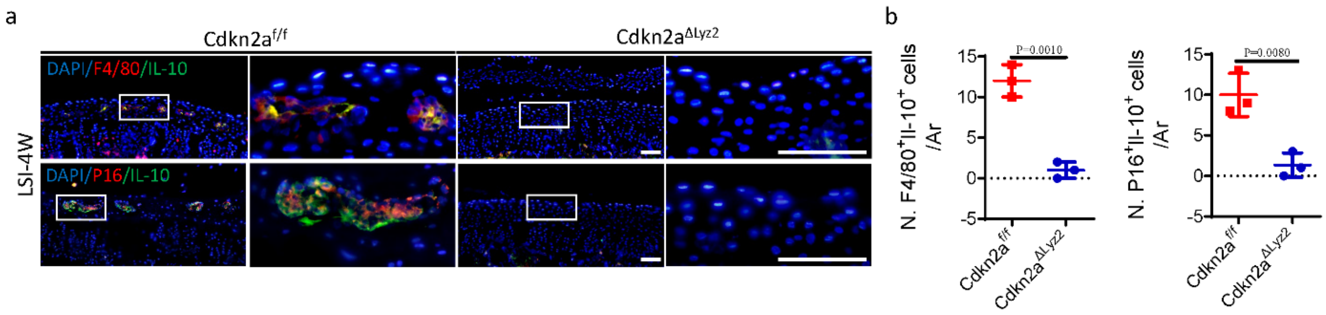

Supplementary Figure 16 Knockout of cdkn2a (p16) in Lyz2^{+} cells decreases the expression of IL-10 (a) Representative co-immunofluorescent images of IL-10 (green) with F4/80 or P16 (red) in mouse caudal endplates of L4/5 in Cdkn2a$^{\Delta Lyz2}$ or Cdkn2a$^{f/f}$ mice at 4 weeks after LSI surgery. Scale bars, 50 µm. (b) Quantitative analysis of (a). Scale bars, 50 µm. n = 3 per group. Data are represented as means ± standard deviations, as determined by two-tailed Student's t-test. Source data are provided as a Source Data file.

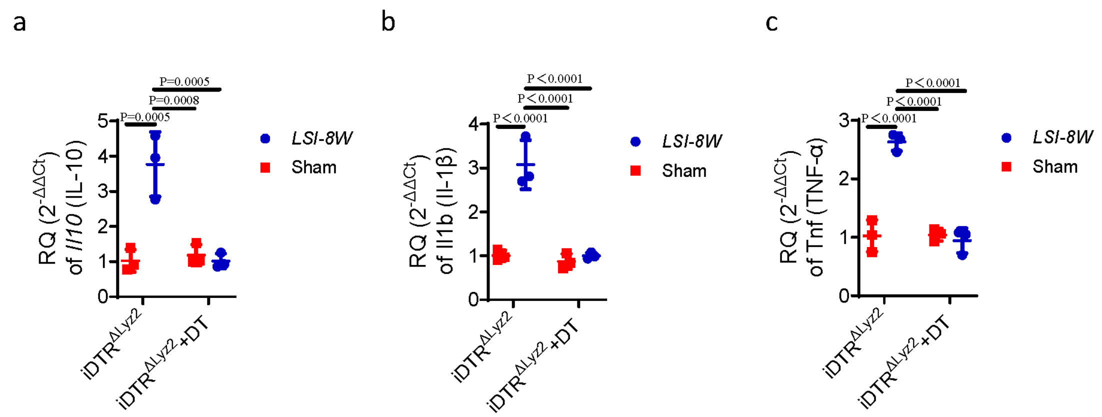

Supplementary Figure 17 The mRNA expression of Il10 (IL-10) (a), Il1b (IL-1β) (b), and Tnf (TNF-α) (c) in the endplates from iDTR$^{\Delta Lyz2}$ mice at 8 weeks after LSI surgery with or without DT injection, determined by qRT-PCR. n = 3 per group. Data are represented as means ± standard deviations, as determined by One-way ANOVA. Source data are provided as a Source Data file.

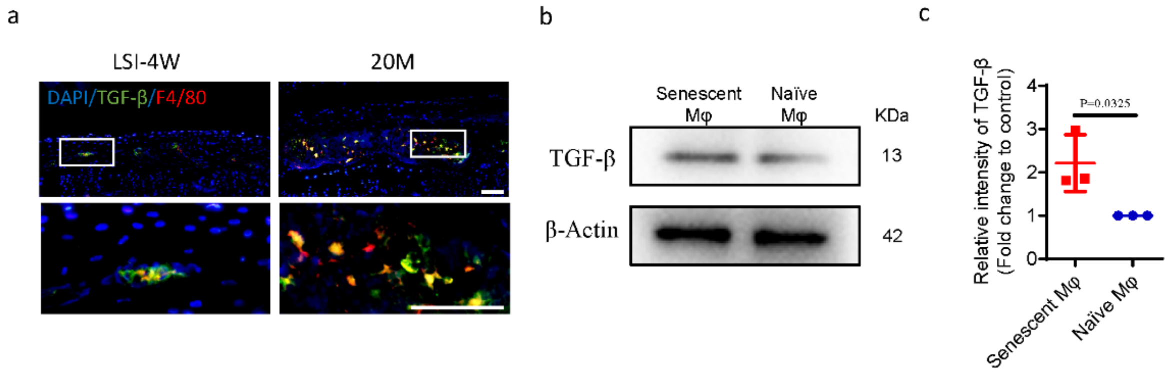

Supplementary Figure 18 TGF-β is elevated in senescent-like macrophages. (a) Representative immunofluorescent images of TGF-β (green), F4/80 (red), and DAPI (blue) staining of nuclei in the endplates of LSI surgery mice or aged mice. Scale bars, 50 μm. (b) Western blot of TGF-β in bone marrow-derived macrophages administrated with 100 μM H_2O_2 or PBS for 4 hours. (c) Quantitative analysis of (b). n = 3 per group. Data are represented as means ± standard deviations, as determined by two-tailed Student's t-test. Source data are provided as a Source Data file.

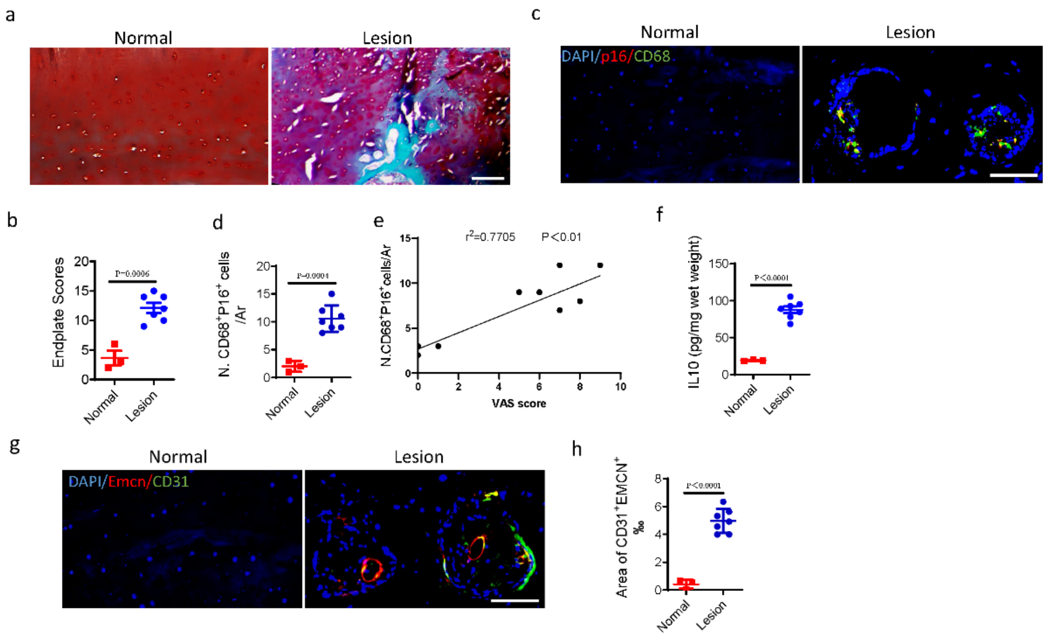

Supplementary Figure 19 CD68$^+$P16$^+$ cells correlate with the sprouting of CD31$^+$Emcn$^+$ blood vessels in human degenerated endplates

(a-g) The endplate samples were harvested from patients without or with endplate lesions. (a) Representative images of safranin O and fast green staining of coronal sections. (b) Endplate scores of (a). (c) Representative immunofluorescent images of CD68 (green), P16 (red) staining, and DAPI (blue) staining of nuclei in the coronal sections of the endplates. (d) Quantitative analysis of the number of P16$^+$CD68$^+$ cells of (c). (e) Correlation analysis of the number of CD68$^+$P16$^+$ cells in endplate tissue with Visual Analogue Scale (VAS) score. (f) ELISA analysis of IL-10 concentration in the lysate of the endplates. (g) Representative immunofluorescent images of CD31 (green), Emcn (red) staining, and DAPI (blue) staining of nuclei in the coronal sections. (h) Permillage of CD31$^+$Emcn$^+$ area in the endplates of (g). Scale bars, 50 μm. n = 3 for normal endplate group, n=7 for endplate lesions group. Data are represented as means ± standard deviations, as determined by two-tailed Student's t-test. Source data are provided as a Source Data file.

Supplementary Table 1 Information for the human samples

	Normal endplate	Lesions in endplate
Sample Size	3	7
Age (Years)	37.3±3.5	64.6±5.4
Sex (Male/Female)	2/1	5/2
Body Mass Index (BMI)	22.3±1.5	23.7±3.1
Disc Level	L4/5 (1)/L5/S1 (2)	L3/4 (1)/L4/5 (2)/L5/S1 (4)
Pfirrmann grading (G3/G4/G5)	1/2	0/3/4

Supplementary Table 2 Primers for ChIP (Endothelial cell, pSTAT3)

	predicted sequence	Forward Primer	Reverse Primer
human-vegfa primer-1	CAGCCTGGAAA, CAGCTAGGAAT	ATAGCCAGGTCAGAAACCA	TCCCTAAGTGCTCCCAAA
human-vegfa primer-2	GAGCCAGGAAA	ACAAGGAGGAAAGTTAGTGGC	CAGCAGCAGGGACAAGGT
human-MMP2 primer-1	TTTCTGAAAAG, CTTTCTGAAAA	TACAGTCAAAGCTGCCCTCT	TTGCCACAGTCCTCACCA
human-MMP2 primer-2	TTTTTGGGAAA	TGTGGCAAACCAAAGTAT	TCCAGCCTGAACAGCATT
human-MMP2 primer-3	CTTCCAGGAAG	CCAGCACTCCACCTCTTT	GTTGGGAACGCCTGACTT
human-PDGFB primer-1	CTTCCAGAAAC	TTTCTTCATGCCTTTCCAC	CCATCTTGCTCCATAATCC

Supplementary Table 3 Primers for qRT-PCR

	Forward Primer	Reverse Primer
Fgf2	AGAAGAGCGACCCTCACATCA	CGGTTAGCACACACTCCTTTG
Vegfa	AGGGCAGAATCATCACGAAGT	AGGGTCTCGATTGGATGGCA
Mmp2	TACAGGATCATTGGCTACACACC	GGTCACATCGCTCCAGACT
Mmp9	TGTACCGCTATGGTTACACTCG	GGCAGGGACAGTTGCTTCT
Cdh5	AAGCGTGAGTCGCAAGAATG	TCTCCAGGTTTTCGCCAGTG
Hif1a	GAACGTCGAAAAGAAAAGTCTCG	CCTTATCAAGATGCGAACTCACA
Pdgfb	CTCGATCCGCTCCTTTGATGA	CGTTGGTGCGGTCTATGAG
Il10	CCCATTCCTCGTCACGATCTC	TCAGACTGGTTTGGGATAGGTTT
Il1b	TTCAGGCAGGCAGTATCACTC	CGTCACACACCAGCAGGTTAT
Tnfα	ATGAGCACAGAAAGCATGA	AGTAGACAGAAGAGCGTGGT
Ccl2	ATTCTGTGACCATCCCCTCAT	TGTATGTGCCTCTGAACCCAC
Il18	CCTACTTCAGCATCCTCTACTGG	AGGGTTTCTTGAGAAGGGGAC
Gapdh	AATGTGTCCGTCGTGGATCTGA	AGTGTAGCCCAAGATGCCCTTC
Cdkn2a	GAGCAGAGGGAGGGAGCAGAAG	GCCGCCTTCGCTCAGTTTCTC
Pecam1	TCCAACAGAGCCAGCAGTATGAG	TCCAATGACAACCACCGCAATG
Sp7	CTCGTCTGACTGCCTGCCTAG	TTGCCTGGACCTGGTGAGATG
Adgre1	TTGGCAAGCATCATGGCATACC	GACGGTTGAGCAGACAGTGAATG
