# Supplementary material for: Senescent-like macrophages mediate angiogenesis for endplate sclerosis via IL-10 secretion in male mice
Source: Nat Commun. 2024 Apr 5;15:2939. doi: 10.1038/s41467-024-47317-1 (PMC10997778; doi:10.1038/s41467-024-47317-1)
Supplement: Supplementary file 3 — Reporting Summary [file 41467_2024_47317_MOESM3_ESM.pdf]

nature portfolio

Corresponding author(s): Shuangfei Ni

Last updated by author(s): Feb 22, 2024

Reporting Summary

Nature Portfolio wishes to improve the reproducibility of the work that we publish. This form provides structure for consistency and transparency in reporting. For further information on Nature Portfolio policies, see our Editorial Policies and the Editorial Policy Checklist.

Statistics

For all statistical analyses, confirm that the following items are present in the figure legend, table legend, main text, or Methods section.

n/a	Confirmed	
☐	☒	The exact sample size (*n*) for each experimental group/condition, given as a discrete number and unit of measurement
☐	☒	A statement on whether measurements were taken from distinct samples or whether the same sample was measured repeatedly
☐	☒	The statistical test(s) used AND whether they are one- or two-sided<br>*Only common tests should be described solely by name; describe more complex techniques in the Methods section.*
☐	☒	A description of all covariates tested
☐	☒	A description of any assumptions or corrections, such as tests of normality and adjustment for multiple comparisons
☐	☒	A full description of the statistical parameters including central tendency (e.g. means) or other basic estimates (e.g. regression coefficient) AND variation (e.g. standard deviation) or associated estimates of uncertainty (e.g. confidence intervals)
☐	☒	For null hypothesis testing, the test statistic (e.g. *F*, *t*, *r*) with confidence intervals, effect sizes, degrees of freedom and *P* value noted<br>*Give P values as exact values whenever suitable.*
☒	☐	For Bayesian analysis, information on the choice of priors and Markov chain Monte Carlo settings
☒	☐	For hierarchical and complex designs, identification of the appropriate level for tests and full reporting of outcomes
☒	☐	Estimates of effect sizes (e.g. Cohen's *d*, Pearson's *r*), indicating how they were calculated

Our web collection on statistics for biologists contains articles on many of the points above.

Software and code

Policy information about availability of computer code

Data collection	The software used for data collection was described in "Methods". No special code were used for data collection.
Data analysis	µCT images were reconstructed and analyzed using NRecon v1.6 and CTAn v1.9 (Skyscan US, San Jose, CA). We analyzed endplate-related parameters using 3-dimensional model visualization software, CTVol v2.0 (Skyscan US). All data analyses were performed using SPSS, version 26.0, software (IBM Corp.). ImageJ (NIH) software was used for quantitative analysis of histology.

For manuscripts utilizing custom algorithms or software that are central to the research but not yet described in published literature, software must be made available to editors and reviewers. We strongly encourage code deposition in a community repository (e.g. GitHub). See the Nature Portfolio guidelines for submitting code & software for further information.

Data

Policy information about availability of data

All manuscripts must include a data availability statement. This statement should provide the following information, where applicable:

- Accession codes, unique identifiers, or web links for publicly available datasets
- A description of any restrictions on data availability
- For clinical datasets or third party data, please ensure that the statement adheres to our policy

All relevant data supporting the findings of this study are available within the article and its Supplementary Information file. Source data are provided with this paper.

Research involving human participants, their data, or biological material

Policy information about studies with human participants or human data. See also policy information about sex, gender (identity/presentation), and sexual orientation and race, ethnicity and racism.

Reporting on sex and gender	Detailed information about the patients and groups is provided in Supplementary Table 1. The gender is not considered in human study design. The sex or gender of patients is determined and collected through the medical record system.
Reporting on race, ethnicity, or other socially relevant groupings	Detailed information about the patients and groups is provided in Supplementary Table 1. All patients were from the First Affiliated Hospital of Zhengzhou University and were Asian.
Population characteristics	Detailed information about the patients and groups is provided in Supplementary Table 1.
Recruitment	Human endplate samples were obtained from patients undergoing spinal surgery in the Department of Orthopaedics at 1st Affiliated Hospital of Zhengzhou University (Zhengzhou, China). There was no self-selection bias in this study.
Ethics oversight	The use of human tissue samples was approved by the Ethics committee of Zhengzhou University. Patients gave written informed consent for the use of the samples.

Note that full information on the approval of the study protocol must also be provided in the manuscript.

Field-specific reporting

Please select the one below that is the best fit for your research. If you are not sure, read the appropriate sections before making your selection.

☒ Life sciences ☐ Behavioural & social sciences ☐ Ecological, evolutionary & environmental sciences

For a reference copy of the document with all sections, see nature.com/documents/nr-reporting-summary-flat.pdf

Life sciences study design

All studies must disclose on these points even when the disclosure is negative.

Sample size	Sample sizes were determined based on our previous experiences and chosen to ensure an adequate statistical power.
Data exclusions	No exclusion criteria were included in this study.
Replication	All experiments were conducted on at least three independent biological replicates and described in the figure legends.
Randomization	All samples and mice were randomly allocated.
Blinding	All samples were evaluated in a blinded manner.

Reporting for specific materials, systems and methods

We require information from authors about some types of materials, experimental systems and methods used in many studies. Here, indicate whether each material, system or method listed is relevant to your study. If you are not sure if a list item applies to your research, read the appropriate section before selecting a response.

Materials & experimental systems

n/a	Involved in the study	
☐	☒	Antibodies
☐	☒	Eukaryotic cell lines
☒	☐	Palaeontology and archaeology
☐	☒	Animals and other organisms
☒	☐	Clinical data
☒	☐	Dual use research of concern
☒	☐	Plants

Methods

n/a	Involved in the study	
☒	☐	ChIP-seq
☐	☒	Flow cytometry
☒	☐	MRI-based neuroimaging

Antibodies

Antibodies used	Histochemistry, immunohistochemistry, and histomorphometry:<br>mouse Endomucin (1:50, sc-65495, Santa Cruz Biotechnology; 1:50, ab106100, Abcam), CD31 (1:100, AF3628, R&D; 1:200, ab76533,

Abcam), F4/80 (1:100, 14-4801-82, eBioscience), γH2A.X (1:100, ab81299, Abcam), p16 (1:100, AF5484, Affinity; 1:400, sc-377412, Santa Cruz Biotechnology), CD68 (1:50, ab955, Abcam; 1:50, 28058-1-AP, Proteintech), IL-10 (1:50, sc-365858, Santa Cruz Biotechnology, Inc.), CD206 (1:50, sc-58986, Santa Cruz Biotechnology, Inc.), iNOS (1:50, sc-7271, Santa Cruz Biotechnology, Inc.), TGFβ (1:50, sc-130348, Santa Cruz Biotechnology, Inc.), RFP (1:250, 5F8, Chromotek GmbH, Planegg-Martinsried, Germany), HMGB1 (1:300, ab18256, Abcam), CGRP (1:100, ab81887, Abcam), osterix (1:200, ab22552, Abcam), and osteocalcin (1:200, M188, Takara)
flow cytometry
Brilliant Violet 421™ anti-mouse F4/80 antibody (1:100, 123131, Biolegend, Inc., San Diego, CA)
Western blotting
Phospho-STAT3 (1:1000, 9145S, CST), STAT3 (1:1000, 10253-2-AP, Proteintech), MMP2 (1:1000, 10373-2-AP, Proteintech), VEGFA (1:1000, 19003-1-AP, Proteintech), PDGFB (1:1000, AF0240, Affinity), GAPDH (1:10000, ET1601-4, HUABIO), P16 (1:1000, AF5484, Affinity), P21 (1:1000, 28248-1-AP, Proteintech), γH2A.X (1:1000, ab81299, Abcam), TGFβ (1:1000, sc-130348, Santa Cruz Biotechnology, Inc.), Bax (1:1000, 50599-2-Ig, Proteintech), Bcl2 (1:1000, 3498S, CST), F4/80 (1:500, 14-4801-82, eBioscience), CD31 (1:1000, sc-376764, Santa Cruz Biotechnology, Inc.), OSX (1:1000, A18699, ABclonal) and β actin (1:5000, 20536-1-AP, Proteintech)

Validation

Histochemistry, immunohistochemistry, and histomorphometry:
mouse Endomucin (1:50, sc-65495, Santa Cruz Biotechnology; 1:50, ab106100, Abcam), CD31 (1:100, AF3628, R&D; 1:200, ab76533, Abcam), F4/80 (1:100, 14-4801-82, eBioscience), γH2A.X (1:100, ab81299, Abcam), p16 (1:100, AF5484, Affinity; 1:400, sc-377412, Santa Cruz Biotechnology), CD68 (1:50, ab955, Abcam; 1:50, 28058-1-AP, Proteintech), IL-10 (1:50, sc-365858, Santa Cruz Biotechnology, Inc.), CD206 (1:50, sc-58986, Santa Cruz Biotechnology, Inc.), iNOS (1:50, sc-7271, Santa Cruz Biotechnology, Inc.), TGFβ (1:50, sc-130348, Santa Cruz Biotechnology, Inc.), RFP (1:250, 5F8, Chromotek GmbH, Planegg-Martinsried, Germany), HMGB1 (1:300, ab18256, Abcam), CGRP (1:100, ab81887, Abcam), osterix (1:200, ab22552, Abcam), and osteocalcin (1:200, M188, Takara)
flow cytometry
Brilliant Violet 421™ anti-mouse F4/80 antibody (1:100, 123131, Biolegend, Inc., San Diego, CA)
Western blotting
Phospho-STAT3 (1:1000, 9145S, CST), STAT3 (1:1000, 10253-2-AP, Proteintech), MMP2 (1:1000, 10373-2-AP, Proteintech), VEGFA (1:1000, 19003-1-AP, Proteintech), PDGFB (1:1000, AF0240, Affinity), GAPDH (1:10000, ET1601-4, HUABIO), P16 (1:1000, AF5484, Affinity), P21 (1:1000, 28248-1-AP, Proteintech), γH2A.X (1:1000, ab81299, Abcam), TGFβ (1:1000, sc-130348, Santa Cruz Biotechnology, Inc.), Bax (1:1000, 50599-2-Ig, Proteintech), Bcl2 (1:1000, 3498S, CST), F4/80 (1:500, 14-4801-82, eBioscience), CD31 (1:1000, sc-376764, Santa Cruz Biotechnology, Inc.), OSX (1:1000, A18699, ABclonal) and β actin (1:5000, 20536-1-AP, Proteintech)

Eukaryotic cell lines

Policy information about cell lines and Sex and Gender in Research

Cell line source(s)

Human umbilical vein endothelial cells (HUVECs) from Procell Life Science&Technology Co. (CP-H082)

Authentication

The suppliers routinely authenticate the cell lines.

Mycoplasma contamination

Mycoplasma test was negative.

Commonly misidentified lines
(See ICLAC register)

No commonly misidentified cell lines were used in this study.

Animals and other research organisms

Policy information about studies involving animals; ARRIVE guidelines recommended for reporting animal research, and Sex and Gender in Research

Laboratory animals

We purchased 4-6 weeks C57BL/6J (WT) male mice from Charles River Laboratories (China). 2- to 19-month-old C57BL/6J (WT) male mice were purchased from Jiangsu Aniphe Biolaboratory Inc for the aging-induced endplate degeneration paradigm. All mice were maintained at the animal facility of Zhengzhou University Animal Experimental Center under 12-hour light/dark cycle at 20-24°C and 45-65% humidity. Animals were housed with a maximum of 5 mice per cage Female mice were only used for breeding. Only male mice were used for further experiments (i.e. histology, etc.).

Wild animals

No wild animals were used in this study.

Reporting on sex

Male mice were used for LSI model and Aging model in this study. The spontaneous osteoporosis that occurs in old female mice could affect the subchondral bone, which is also an important characteristic in cartilage pathology. To avoid this, we use male mice in our study, according other similar studies.

Field-collected samples

The study did not involve samples collected from the field.

Ethics oversight

All mice were maintained at the animal facility of Zhengzhou University Animal Experimental Center. All animal experiments were approved by the Animal Care and Use Committee of Zhengzhou University Animal Facility and in compliance with the relevant laws. All animal experiments complied with the ARRIVE guidelines for reporting animal experiments.

Note that full information on the approval of the study protocol must also be provided in the manuscript.

Plants

Seed stocks

Report on the source of all seed stocks or other plant material used. If applicable, state the seed stock centre and catalogue number. If plant specimens were collected from the field, describe the collection location, date and sampling procedures.

Novel plant genotypes

Describe the methods by which all novel plant genotypes were produced. This includes those generated by transgenic approaches, gene editing, chemical/radiation-based mutagenesis and hybridization. For transgenic lines, describe the transformation method, the number of independent lines analyzed and the generation upon which experiments were performed. For gene-edited lines, describe the editor used, the endogenous sequence targeted for editing, the targeting guide RNA sequence (if applicable) and how the editor was applied.

Authentication

Describe any authentication procedures for each seed stock used or novel genotype generated. Describe any experiments used to assess the effect of a mutation and, where applicable, how potential secondary effects (e.g. second site T-DNA insertions, mosiacism, off-target gene editing) were examined.

Flow Cytometry

Plots

Confirm that:

☒ The axis labels state the marker and fluorochrome used (e.g. CD4-FITC).

☒ The axis scales are clearly visible. Include numbers along axes only for bottom left plot of group (a 'group' is an analysis of identical markers).

☒ All plots are contour plots with outliers or pseudocolor plots.

☒ A numerical value for number of cells or percentage (with statistics) is provided.

Methodology

Sample preparation

Detailed information is provided in "Cell sorting and flow cytometry analysis of cartilage endplate" of "Methods"

Instrument

BD LSRFortessa flow cytometer

Software

FlowJo software (version 10, BD Bioscience)

Cell population abundance

At least 5000 cells were acquired for each sample.

Gating strategy

The Fluorescence Minus One Control were used to define the boundaries between positive and negative cell populations inmutiple-luorochromes panels. Viable cells were selected based on fsc/ssc. The following gating strategies were used: P16 +F4/80 cells were gated as F4/80+ tdTom+.

☒ Tick this box to confirm that a figure exemplifying the gating strategy is provided in the Supplementary Information.
